# Entrapment of the Impella device by the guiding catheter: a cautionary pitfall of Impella-protected percutaneous coronary intervention

**DOI:** 10.1093/ehjcr/ytae096

**Published:** 2024-02-19

**Authors:** Hiroyuki Yamamoto, Nobuhiro Watanabe, Koki Matsuo, Tomofumi Takaya

**Affiliations:** Division of Cardiovascular Medicine, Department of Internal Medicine, Hyogo Prefectural Harima-Himeji General Medical Center, 3-264 Kamiya-cho, Himeji 670-8560, Japan; Division of Cardiovascular Medicine, Department of Internal Medicine, Hyogo Prefectural Harima-Himeji General Medical Center, 3-264 Kamiya-cho, Himeji 670-8560, Japan; Division of Cardiovascular Medicine, Department of Internal Medicine, Hyogo Prefectural Harima-Himeji General Medical Center, 3-264 Kamiya-cho, Himeji 670-8560, Japan; Division of Cardiovascular Medicine, Department of Internal Medicine, Hyogo Prefectural Harima-Himeji General Medical Center, 3-264 Kamiya-cho, Himeji 670-8560, Japan; Department of Exploratory and Advanced Research in Cardiology, Kobe University Graduate School of Medicine, 7-5-1 Kusunoki-cho, Chuo-ku, Kobe 650-0017, Japan

## Case description

**Figure 1:**
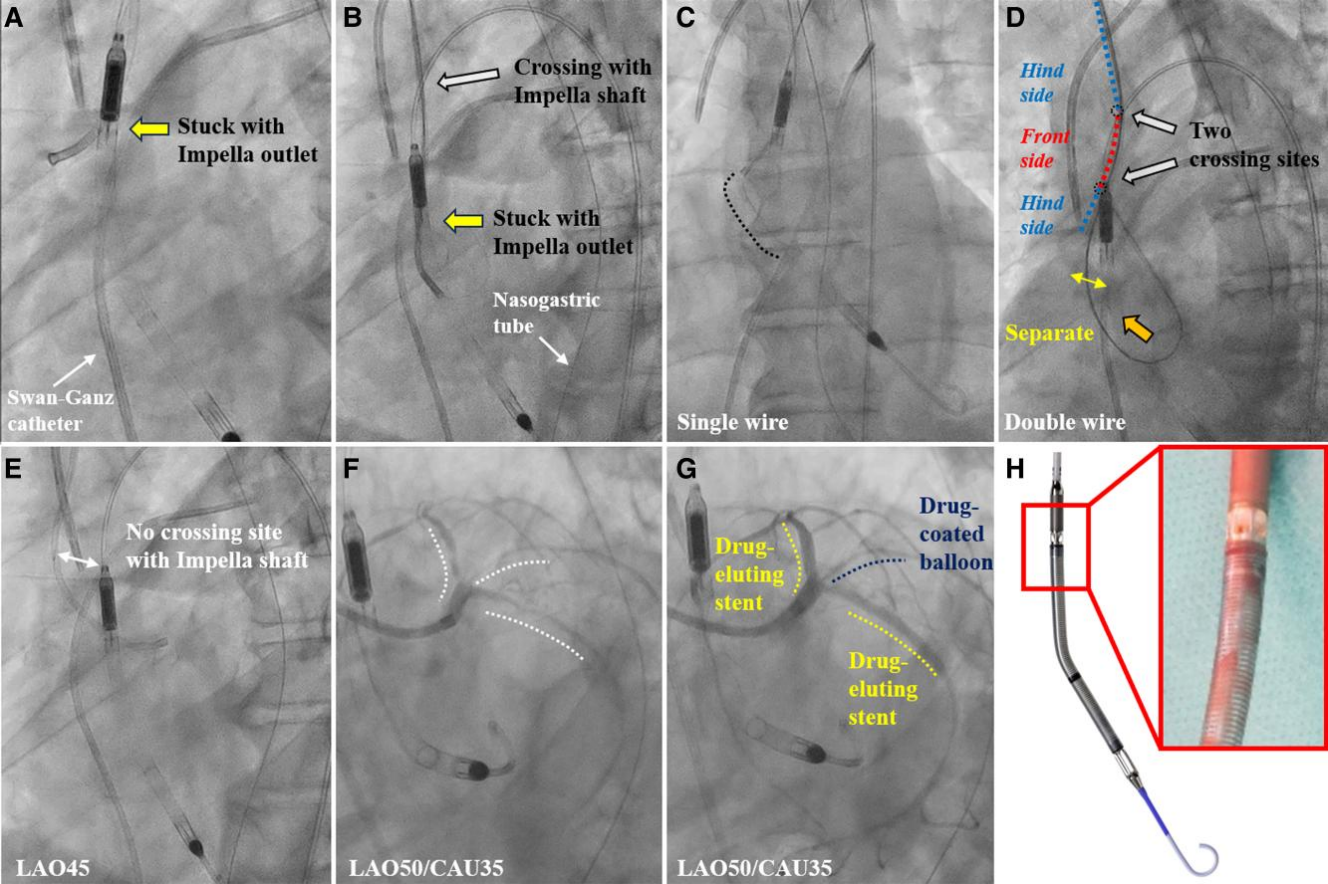
(*A*, *B*) Angiograms showing the positional relationship between the guiding catheter and the Impella. The Impella device moves upward and downward with the guiding catheter motion, suggesting entrapment of the guiding catheter with the Impella outlet. (*C*) The interaction between the guiding catheter and Impella outlet is not improved using a single wire. (*D*) The guiding catheter separates from the Impella outlet when double wires are pushed onto the aortic cusp. (*E*) No interaction between the Impella device and Judkins left guiding catheter. (*F*) Before and (*G*) after percutaneous coronary intervention. White dotted line shows stenosis. (*H*) Magnified view showing the windows of the Impella outlet.

A 67-year-old man presented with acute decompensated heart failure and cardiogenic shock. Transthoracic echocardiography revealed severe global left ventricular hypokinesis with a left ventricular ejection fraction of 25%. Emergency coronary angiography revealed multiple stenoses without obstruction. Therefore, transfemoral left ventricular assist (Impella CP, Abiomed, USA) support was performed to increase coronary flow through diastolic augmentation. However, low cardiac output syndrome made removing the Impella device difficult, requiring percutaneous coronary revascularization. Although a backup guiding catheter (GC; Hyperion SPB3.5, Asahi Intecc, Japan) was initially used via the right radial artery, cannulating the coronary artery was difficult. After manipulating the GC clockwise, the Impella device moved upwards with the GC motion, suggesting entrapment of the GC with the Impella outlet (*Figure 1A* and *B*; Supplementary material online, *Video S1*). We pushed the GC to the cardiac side and attempted to extract the GC using a single 0.035 inch conventional wire; however, it was difficult because of the interaction. Next, manually adjusting the Impella shaft’s position via the femoral artery while pushing the 0.035 inch Amplatz Extra-Stiff guidewire (Cook, USA) inserted into the GC as double wires against the aortic cusp disengaged the entrapment (*Figure 1C* and *D*). Subsequent percutaneous coronary intervention (PCI) using a non-backup type GC (Hyperion JL3.5, Asahi Intecc, Japan) was successfully performed without interaction with the Impella, and the patient recovered from low cardiac output syndrome (*Figure 1E–G*).

Impella effectively stabilizes haemodynamics in patients with cardiogenic shock and complex high-risk PCI.^1^ Impella outlet comprises five windows that could interact with other devices (*Figure 1H*).^2^ The positional relationship between the Impella outlet and GC should be considered when manipulating GC in the ascending aorta. To avoid entrapment of the GC and Impella device, we suggest inserting the GC from a coronary access route not crossing the Impella device and avoiding manipulation of the GC without turning it drastically. This case demonstrated that PCI via the right upper limb may result in the GC crossing the Impella, potentially causing device interference and Impella dislocation.

## Supplementary Material

ytae096_Supplementary_Data

## Data Availability

The data underlying this article are available in the article and its online Supplementary material.

## Supplementary material

**Consent:** In accordance with the COPE guidelines, informed consent was obtained from the patient for his participation in this study and the publication of the case and accompanying images.

**Funding:** None declared.

## References

[1] Fujimoto Y, Sakakura K, Fujita H. Complex and high-risk intervention in indicated patients (CHIP) in contemporary clinical practice. Cardiovasc Interv Ther 2023;38:269–274. 36971962 10.1007/s12928-023-00930-1

[2] Sharma A, Bertog S, Mbai M. Impella placement across transcatheter aortic valves: a potential for device-device interaction. JACC Cardiovasc Interv 2020;13:2574–2575. 32861634 10.1016/j.jcin.2020.05.047

